# Development of a High-Sensitivity Optical Accelerometer for Low-Frequency Vibration Measurement

**DOI:** 10.3390/s18092910

**Published:** 2018-09-01

**Authors:** Rui-Jun Li, Ying-Jun Lei, Zhen-Xin Chang, Lian-Sheng Zhang, Kuang-Chao Fan

**Affiliations:** 1 School of Instrument Science and Opto–Electronics Engineering, Hefei University of Technology, Hefei 230009, China; rj-li@hfut.edu.cn (R.-J.L.); 2016170008@mail.hfut.edu.cn (Y.-J.L.); 2017110054@mail.hfut.edu.cn (Z.-X.C.); fan@dlut.edu.cn (K.-C.F.); 2 School of Mechanical Engineering, Dalian University of Technology, Dalian 116024, China

**Keywords:** optical accelerometer, low-frequency vibration, leaf spring, four-quadrant photodetector

## Abstract

Low-frequency vibration is a harmful factor that affects the accuracy of micro/nano-measuring machines. Low-frequency vibration cannot be completely eliminated by passive control methods, such as the use of air-floating platforms. Therefore, low-frequency vibrations must be measured before being actively suppressed. In this study, the design of a low-cost high-sensitivity optical accelerometer is proposed. This optical accelerometer mainly comprises three components: a seismic mass, a leaf spring, and a sensing component based on a four-quadrant photodetector (QPD). When a vibration is detected, the seismic mass moves up and down due to the effect of inertia, and the leaf spring exhibits a corresponding elastic deformation, which is amplified by using an optical lever and measured by the QPD. Then, the acceleration can be calculated. The resonant frequencies and elastic coefficients of various seismic structures are simulated to attain the optimal detection of low-frequency, low-amplitude vibration. The accelerometer is calibrated using a homemade vibration calibration system, and the calibration experimental results demonstrate that the sensitivity of the optical accelerometer is 1.74 V (m·s^−2^)^−1^, the measurement range of the accelerometer is 0.003–7.29 m·s^−2^, and the operating frequencies range of 0.4–12 Hz. The standard deviation from ten measurements is under 7.9 × 10^−4^ m·s^−2^. The efficacy of the optical accelerometer in measuring low-frequency, low-amplitude dynamic responses is verified.

## 1. Introduction

In ultra-precision measurement, ultra-precision machining, metrological verification and similar applications, the low-frequency micro-vibration caused by human activity and natural factors has great influence. Its amplitude is extremely small (in the micron range) and it cannot be eliminated completely by passive control methods, such as the use of air-floating platforms. Thus, a vibration control technique is a good solution. The main problems of active vibration isolation techniques involve the design of appropriate sensor techniques and control schemes. In terms of vibration frequency, the vibration generated by certain human activities, transportation, and mechanical devices ranges of 1–10 Hz [1,2]. Accordingly, the accelerometer used in measuring environmental vibration must be designed specifically for low frequency and high sensitivity.

Table 1 shows a summary of the existing accelerometers for low-frequency, low-amplitude vibration, which include piezoelectric (PZT) accelerometers, strain accelerometers, fiber Bragg grating (FBG) accelerometers, micro-electro-mechanical system (MEMS) accelerometer, optical accelerometers and other types.

Tian et al. [3] designed a high sensitivity and low transverse effect PZT accelerometer. The piezoelectric accelerometer fabricated on an n-type single crystal silicon wafer and the sensor chips were wire-bonged to printed circuit boards (PCBs). The sensitivity of the accelerometer is 9 mV/g, the linearity is 0.0205, and the hysteresis error is 0.0033. Zou et al. [4], Nishshanka et al. [5], and Tims et al. [6] have also made progress in this field and the detailed data is shown in Table 1. From Table 1, we can see that the PZT accelerometers have a wide bandwidth. However, their sensitivities are low, and they cannot be used to detect low frequency micro-vibrations.

Kamentse et al. [7] presented a strain gauge accelerometer. A nonconductive ceramic beam was used as elastic element. Four strain gauges were attached on the surface of the beam using screen printing process. This accelerometer has a wide detection range. Santana et al. [8] have also made progress in this field and the detailed data is shown in Table 1. Strain gauge accelerometers have widely used in industry because of high stability and low price. However, the accuracy of the strain gauge accelerometer is low, and the detection of low frequency micro-vibration cannot be achieved.

Liu et al. [9] applied FBG to an accelerometer. In their design, two symmetrical bended spring plates were used as elastic elements so as to double the wavelength shift of the FBG. This accelerometer has a low-frequency response range of 0.7–20 Hz. However, it cannot describe the output when the outside acceleration is small (less than 1 m·s^−2^). Gao et al. [10], Zhang et al. [11], Zhang et al. [12], Zeng et al. [13], and Li et al. [14] have also made progress in this field and their results is shown in Table 1. It can be seen that the FBG accelerometers can achieve the detection of low-frequency vibration. However, it is difficult for them to detect the vibration of low-frequency and low-amplitude since their resolution is limited.

Sabato et al. [15,16,17] developed a wireless MEMS accelerometer for micro-vibrations. This accelerometer used voltage to frequency conversion (V/F) other than analog to digital conversion (ADC) to promote the accelerometer’s performance. However, the validations for the wireless accelerometer in the low-frequency, ultra-low amplitude range have not been reported. Swartz et al. [18], Cho et al. [19], Whelan et al. [20] Meyer et al. [21], Rice et al. [22], and Kohler et al. [23,24] have also made progress in this field and their results are shown in Table 1. We can find that the MEMS accelerometer has a wide frequency response range. However low-frequency and low-amplitude vibration detection cannot be achieved because their low sensitivity.

Several accelerometers were developed based on the high precision displacement or angle sensor modified from DVD pick up head [25,26,27]. These optical accelerometers have high sensitivity and enable low amplitude vibration detection. The cantilever beam produces angular motion when sensing vibration, thereby causing measurement errors.

In addition to the above technologies, Zheng et al. [28] proposed a new maglev sensor to measure ultra-low frequency (ULF) vibration, which uses hybrid-magnet levitation structure with electromagnets and permanent magnets as the supporting component. Jiang et al. [29] proposed an all-metal double metal diaphragm-based optical fiber accelerometer with low transverse sensitivity. Lin et al. [30] designed a compact-size fiber optic accelerometer to achieve both high resolution and wide dynamic range.

In summary, for the cost-effective and accurate measurement of low-frequency, low-amplitude vibration, a new optical accelerometer system is developed in this study. A centrally symmetric leaf spring fixed on a seismic mass was used as a vibration sensitive unit, four-quadrant photodetector (QPD) based optical sensor was used to sensor the seismic mass’s displacement. Section 2 introduces the structure and principles. Section 3 explains the design, analysis, and fabrication of the optical accelerometer. The performances of the optical accelerometer are discussed in Section 4. The summary and prospect of the optical accelerometer are presented in Section 5.

## 2. Structure and Principle

**Figure 1:**
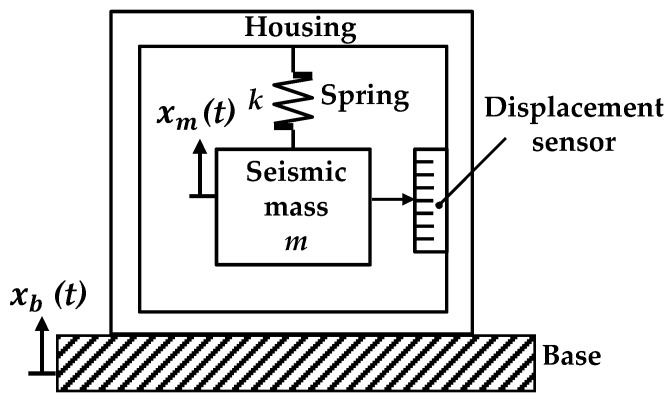
Typical accelerometer structure diagram.

As shown in Figure 1, a common contact accelerometer comprises a spring, a seismic mass, and a displacement sensor arranged within a housing attached to a base. In operation, the base is mounted on the vibrating structure to be measured, and the relative displacement between the seismic mass and the base is recorded by the displacement sensor. Following Newton’s second law, the force acting on the seismic mass *m* can be expressed as:

(1) $$ma=k(x_{m}-x_{b}),$$

where *x_m_* is the displacement of the seismic mass and *x_b_* is the displacement of the base; thus, the relative displacement between the seismic mass and the base can be expressed as:

(2) $$(x_{m}-x_{b})=\frac{m}{k}a.$$

If the acceleration *a* is constant, then the larger the seismic mass *m* and the smaller the elastic coefficient of spring *k* would result in the larger relative displacement between the seismic mass and the base. Therefore, the stronger the output signal of the displacement sensor is, the higher the sensitivity of the accelerometer will be. Nevertheless, if the seismic mass *m* is too heavy and the elastic coefficient *k* of the spring is too low, then the seismic mass is liable to generating a lateral yaw and increasing the measurement error. Thus, the elastic coefficient *k* of the elastic support and the mass *m* of the seismic mass directly affect the accelerometer performance. The appropriate spring and seismic mass must be selected to realize the high-sensitivity detection of low-frequency vibration. The third part of this paper emphasizes the design of spring and seismic mass.

**Figure 2:**
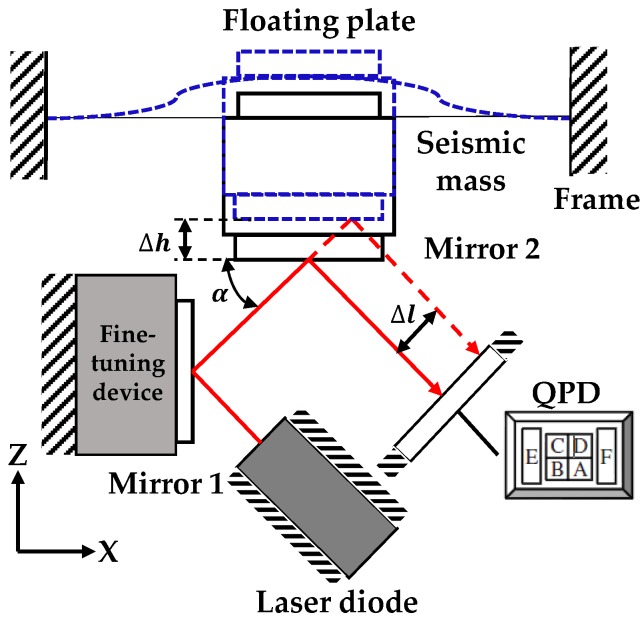
The schematic of an optical accelerometer. QPD: four-quadrant photodetector.

On the basis of the typical accelerometer structure shown in Figure 1, a leaf spring is used as the elastic support in this study. As shown in Figure 2, the leaf spring is mounted on the frame of the accelerometer. A seismic mass fixed to the lower surface of the leaf spring is used as a vibration system, and the QPD in association with a laser diode is regarded as a pick-up sensor. Mirror 2 is attached to the bottom of the seismic mass. When vibration occurs, the leaf spring undergoes elastic deformation, and the seismic mass moves up and down. The position of the projected light spot on the four-quadrant photodetector then shifts. To add a fine-tuning device for optical path adjustment, Mirror 1 is added on the left side and installed on the fine-tuning device.

Figure 2 shows the optical path of the sensor system. The laser beam from the laser diode with an incident angle is reflected by the mirrors and then projected onto the QPD. A vertical displacement ∆*h* of Mirror 2 causes a lateral shift ∆*l* of the light spot on the QPD. The relationship between ∆*h* and ∆*l* is expressed by:

(3) $$\Delta l=\frac{\Delta h}{\sin\alpha },$$

where *α* is the angle between the laser and Mirror 2. Equation (3) shows that the vertical motion of the seismic mass is magnified by the factor of (sin*α*)^−1^. Provided that the value of angle *α* is smaller, the sensitivity of the sensor is higher, but this situation also increases the lateral size of the accelerometer. Considering the elastic structure, optical path, and circuit design, which affect accelerometer sensitivity, the existing amplifier design can meet the requirements well. Therefore, in the design of the elastic structure and optical path, although the basic performance requirements of the accelerometer are met, we must consider the difficulty of processing and reduce the cost. Thus, 45° is selected as the value of the included angle *α*. Mirror 1, which is introduced into the design to adjust the optical path, is mounted on a 2D fine-tuning device, and the angle can be fine-tuned.

## 3. Design, Analysis and Fabrication

In this study, the accelerometer is intended to measure the movement of the environmental vibration (1–10 Hz) [1,2]. The frequency of the acceleration cannot be correctly measured until it is lower than 20% of the resonance frequency of the accelerometer when the acceleration acts on the base. This limits the frequency response design of the accelerometer. Therefore, the required resonance frequency value of the accelerometer should be approximately 50 Hz. In addition to the resonance frequency of the accelerometer, the elastic coefficient of the accelerometer’s elastic part must be considered in the design.

**Figure 3:**
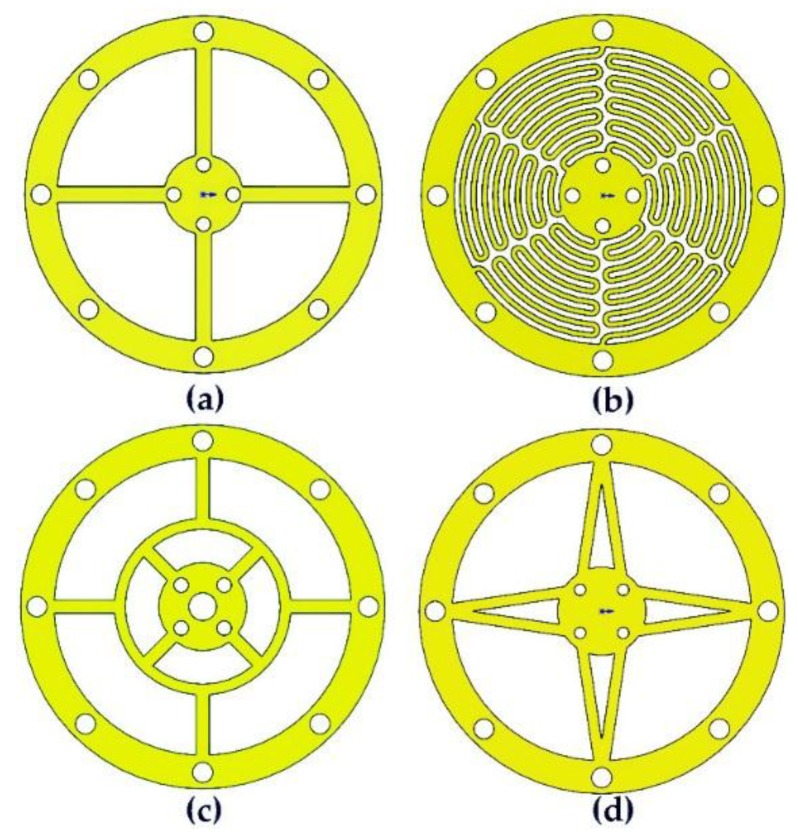
Leaf spring shapes. (**a**) cross; (**b**) serpentine; (**c**) double circular; (**d**) V shape.

**Figure 4:**
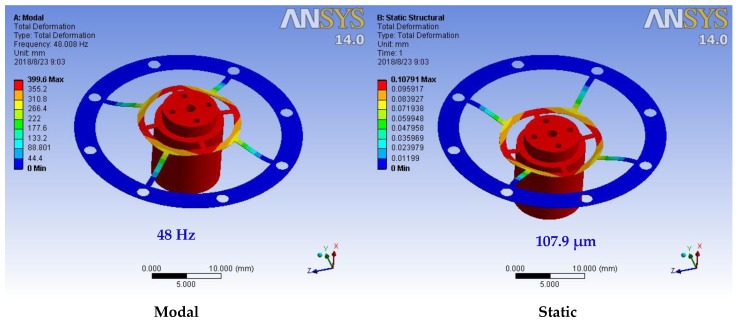
The modal and static structural analysis result of simulation software ANSYS (shape c).

**Figure 5:**
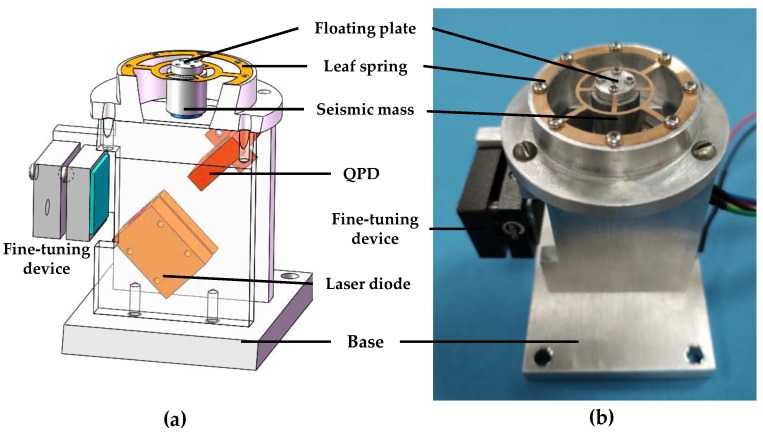
Optical accelerometer (**a**) internal schematic, (**b**) photo.

As shown in Figure 2, the elastic part of the optical accelerometer contains a seismic mass, a leaf spring, a floating plate, and a mirror. The symmetrical structure has the same deformation; thus, the horizontal position of Mirror 1 is maintained to avoid the lateral yaw motion. The design of the leaf spring must consider its structural symmetry and elasticity coefficient. Given these two factors and previous experience in leaf spring design, we propose four types of leaf springs (as shown in Figure 3). The leaf spring of shape (a) has a simple structure and a large elastic coefficient. The leaf spring of shape (b) has a complex structure but a small elastic coefficient. The leaf springs of shapes (c) and (d) are simpler than that of shape (b), and the elasticity coefficient is more moderate than those of (a) and (b). The outer rings of the four types of leaf springs are the same size (33 mm). In accordance with the laboratory’s existing leaf spring manufacturing process technology, leaf spring of two thicknesses (0.1 and 0.15 mm) are produced. To reduce the processing difficulty of the accelerometer, a 10 mm diameter cylindrical structural steel block is selected as the seismic mass. Its mass is 6.25 g. Simulation software ANSYS 14.0 (ANSYS Co., Ltd., Pittsburgh, PA, USA) is used to simulate the eight seismic structures. The simulation results are shown in Table 1, in which the elastic coefficient *k* is calculated by:

(4) $$k=\frac{mg}{\Delta x},$$

where *m* is the mass of the seismic mass, *g* is the acceleration of gravity, and ∆*x* is the static deformation of the seismic structure. Following the results of the analysis in Table 2, the two seismic structures formed by the leaf spring of shape (a) have resonance frequencies higher than the design requirements; however, their elasticity coefficient is large, which may reduce the sensitivity of the accelerometer. The resonant frequencies of the two types of seismic structures that compose the leaf spring of shape (b) are much lower than the design requirements, and the first three resonant frequencies have a small difference, which can easily generate higher-order resonance and cannot be used in accelerometers. The problem of the two types of seismic structures formed by the leaf spring of shape (d) is similar to that of the structure of the leaf spring of shape (a). The most appropriate seismic structure consists of the leaf spring with (c) and a thickness of 0.1 mm, in which the resonant frequency of the seismic structure is 48 Hz, the static deformation is 107.9 μm, and the elastic coefficient is 0.56 mN/μm, as shown in Figure 4. This seismic structure has a modest rigidity and can satisfy the requirements of the seismic structure in this study. Thus, the leaf spring with shape (c) and 0.1 mm thickness is selected for fabrication. The leaf spring is fabricated from beryllium bronze, and the outer and inner diameters are specified as 33 and 16 mm, respectively. The seismic mass is fabricated from 4041 alloy steel. The dimensions of the completed optical accelerometer are as follows: length, 70 mm; width, 50 mm; and height, 70 mm. Figure 5 presents a photograph of the completed optical accelerometer. The laser diode and the QPD are fixed to the accelerometer frame by a fixing seat. Mirror 1 is attached to the fine-tuning device, which is fixed to the accelerometer frame by screws. As shown in Figure 5, in order to reduce optical system noise, the optical part of the proposed accelerometer is sealed in a housing shell, which can avoid the disturbance from an external light source.

The model of the QPD we used in the accelerometer is SPOT-4D (OSI Optoelectronics Co., Ltd., Hawthorne, CA, USA), which has an active area of 1.3 mm × 1.3 mm, a responsivity of about 0.4 A W^−1^ corresponding with a wave length of 630 nm, a maximum dark current of 1 nA, and a noise equivalent power (NEP) of 8.7 × 10^−15^ W (Hz^1/2^)^−1^. The four-channel photocurrent signal output (μA) by the QPD is converted into a voltage signal (using a resistor of 33 kΩ) output by a low-power quad-operational amplifier LM124 (Texas Instruments Co., Ltd., Dallas, TX, USA). A low-pass filter circuit with a cut-off frequency of 40 Hz was used to reduce the interference from high frequency electrical noise and the power supply.

## 4. Experimental Setup and Measurement Results

In general, the accelerometer is calibrated by the recording of its output under a particular excitation force and then a comparison of this output with that generated by a reference accelerometer under the same excitation conditions [26]. This type of calibration is known as the comparative method. In this study, a laser displacement sensor (LDS) (OptoNCDT 1402-10, MICRO-EPSILON Co., Ltd., Ortenburg, Germany) is applied as the reference sensor. The measurement range is 10 mm, the resolution is 1 μm, and the bandwidth is 1.5 kHz. The displacement values from the LDS can be converted to acceleration by:

(5) $$a={\left(2\pi f\right)}^{2}D,$$

where *D* is the measured amplitude of vibration by LDS and *f* is the frequency of the sinusoidal excitation signals applied to the calibration vibration generator.

**Figure 6:**
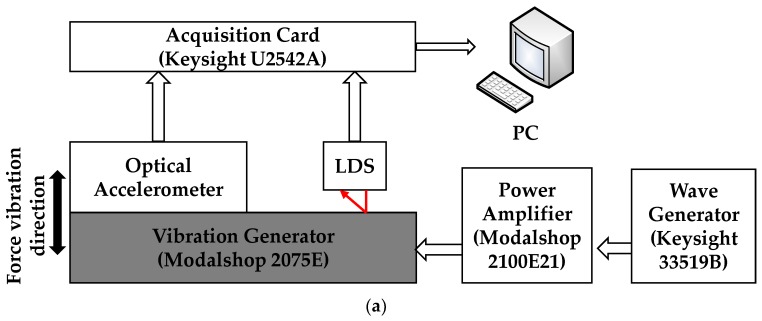

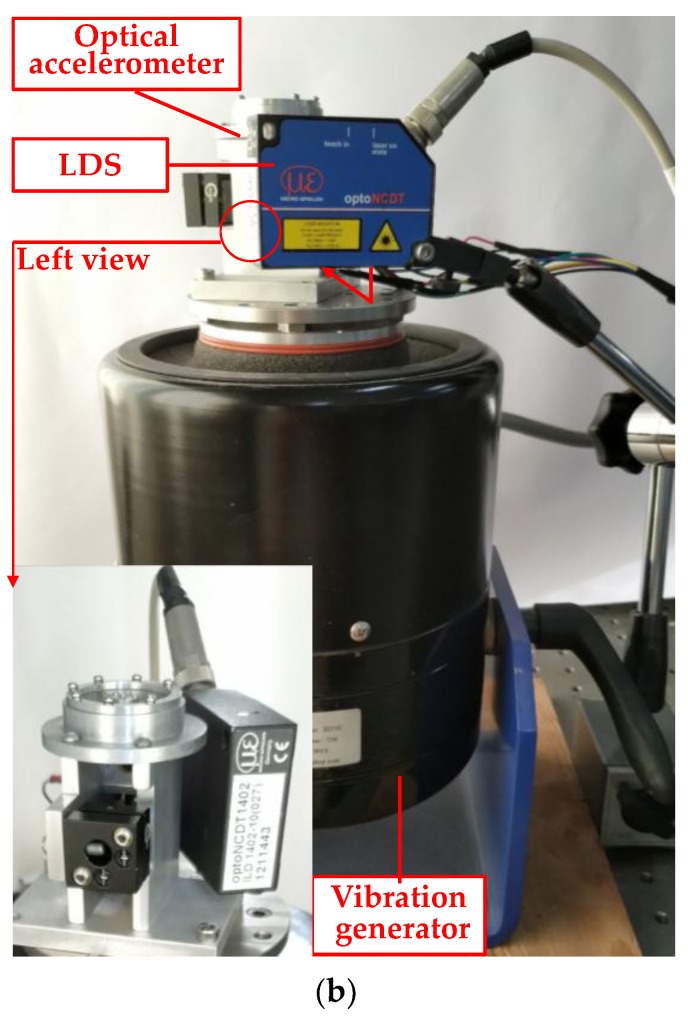
Experimental setup for the designed optical accelerometer calibration. (**a**) Diagram, (**b**) photo. LDS: laser displacement sensor.

Figure 6 illustrates the experimental setup used for the comparison calibration test. In this arrangement, a vibration generator (Modalshop 2075E, The Modalshop MTS Systems Co., Ltd., Cincinnati, OH, USA) was used to provide the excitation acceleration. A high-precision wave generator (Keysight 33519B, Keysight Technologies Co., Ltd., Santa Rosa, CA, USA) was used to generate the sinusoidal excitation signal, which was then amplified by a power amplifier (Modalshop 2100E21, The Modalshop MTS Systems Co., Ltd., Cincinnati, OH, USA) to drive the vibration generator. The output signals of the proposed accelerometer and LDS were acquired to the computer by a synchronous acquisition card (Keysight U2542A, Keysight Technologies Co., Ltd., Santa Rosa, CA, USA).

### 4.1. Resonance Frequency Measurement

**Figure 7:**
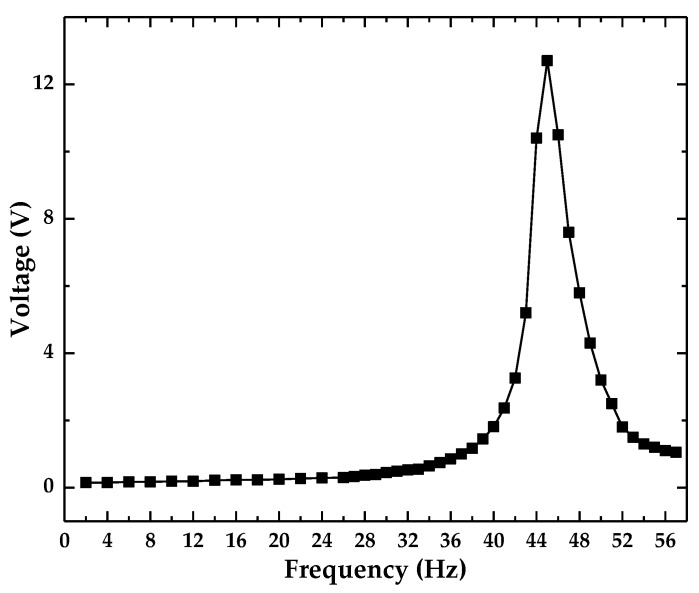
Frequency response test of the designed optical accelerator.

The resonance frequency of the optical accelerometer was obtained using the experimental set-up shown in Figure 6. The wave generator was used to output a swept sine waveform signal, which was input to the power amplifier, amplified, and then supplied to the vibration generator. The outputting voltage from the optical accelerometer was recorded in the range of 2–26 Hz with an interval of 2 Hz, and in the range of 26–57 Hz with an interval of 1 Hz. The results are presented in Figure 7. 

The first resonance frequency of the optical accelerometer is approximately 45 Hz, which is in agreement with the simulation results obtained by ANSYS 14.0 (Figure 5). The difference is mainly due to the machining and the overall assembly errors of the leaf spring and the accelerometer.

### 4.2. Acceleration Sensitivity Measurement at Low-Frequency

**Figure 8:**
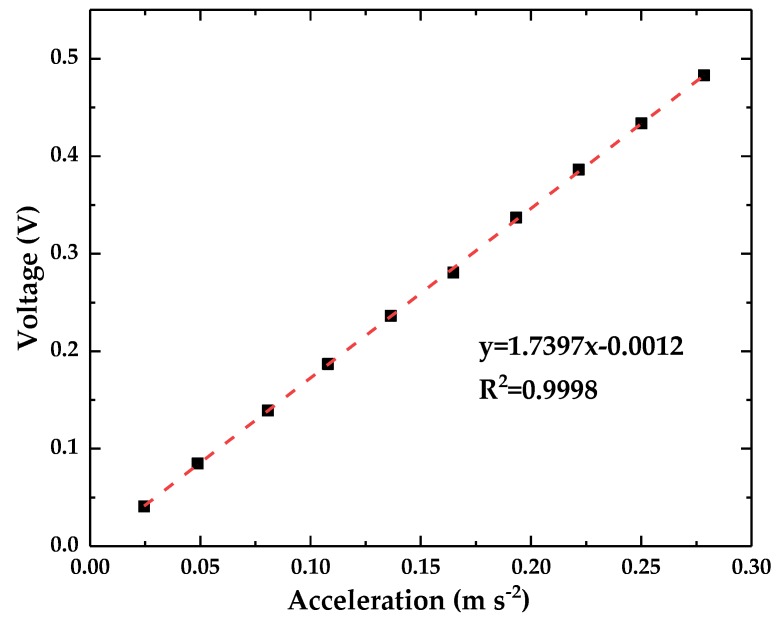
The sensitivity of the optical accelerometer.

The acceleration sensitivity of the optical accelerometer was measured at frequency far lower than the resonance frequency, which can make sure that the proposed accelerometer works steadily. A sine waveform with a constant frequency of 5 Hz was generated to drive the vibration generator with the amplitude changing from 1 Vpp to 10 Vpp. The measured displacement amplitude by LDS changes from 24 μm to 282 μm. The converted output acceleration signal of the LDS and the output voltage of the optical accelerometer are presented in Figure 8. The results from data fitting indicated that the sensitivity of the optical accelerometer is 1.739 V (m·s^−2^)^−1^. The R-square is 0.9998, thereby indicating that the optical accelerometer has excellent linearity characteristics.

**Figure 9:**
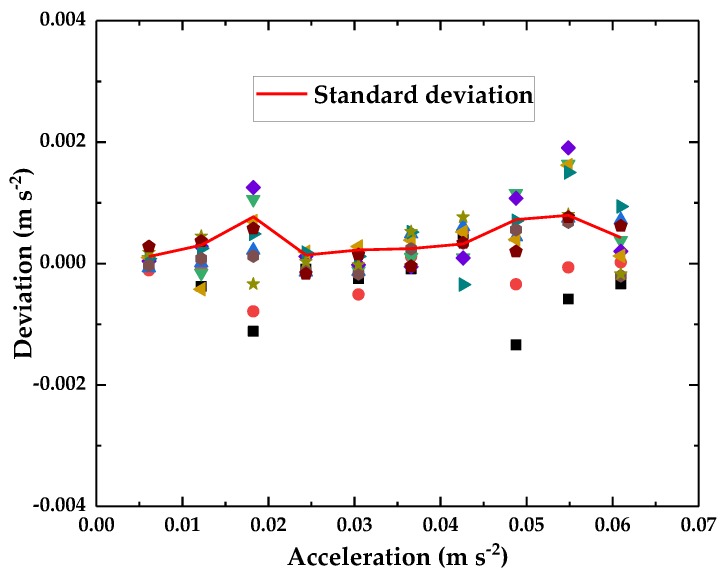
The stability of the optical accelerometer. (The points with same shape and color are the results of the same measurement).

To test the stability of the accelerometer’s output, repetitive tests were performed. A homemade high-performance vibration generator [31] is used to provide an acceleration excitation signal. The vibration generator can achieve steady output displacement with frequency range of 0.6–50 Hz, an analytical displacement resolution of 3.1 nm and an acceleration range of 0–1.93 m·s^−2^. In order to monitor the displacement of the homemade vibrator, an eddy current sensor [32,33] is used as a reference sensor, the output sensitivity of the sensor is 0.2312 V·μm^−1^, the measurement range is 50 μm, the resolution is 0.72 nm, and the maximum nonlinearity error is less than 1%. We used a sine waveform with a constant frequency of 8 Hz as the excitation signal and varied the input amplitude of the vibration generator within the range of 12–120 Vpp (corresponding amplitude ranging of 2.2–19.7 μm). The reference sensor and optical accelerometer output signals were recorded. The experiment was repeated ten times. The output voltage of the optical accelerometer was converted into the corresponding acceleration by using the sensitivity value obtained from Figure 8. As shown in Figure 9, the deviation between the measured acceleration and the reference acceleration served as the ordinate, and the excitation acceleration served as the abscissa. The standard deviation of ten measurements ranges of 1.4 × 10^−4^–7.9 × 10^−4^ m·s^−2^.

### 4.3. Frequency Response Range, Acceleration Detection Range and Phase-Frequency Response

**Figure 10:**
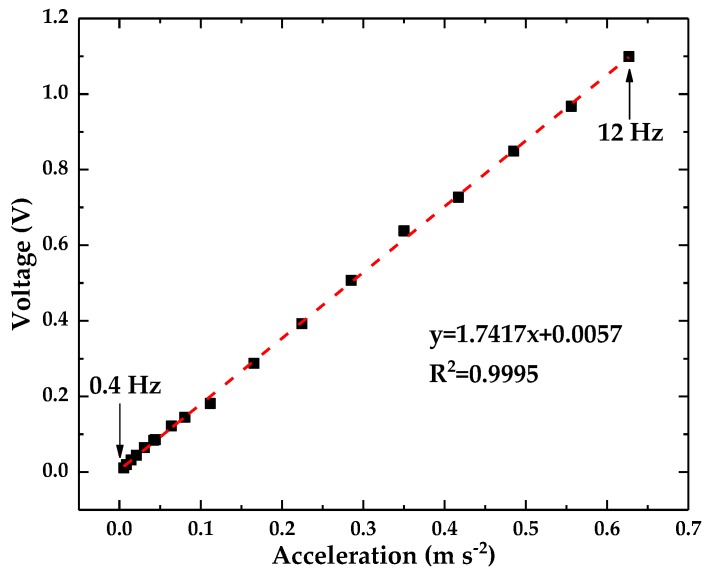
Frequency response range of the optical accelerometer.

The frequency response of the proposed optical accelerometer was tested using the setup in Figure 6. The wave generator was used to output sine waveform signals with frequencies in the range of 0.4–1 Hz with an interval of 0.1 Hz, and in the range of 1–12 Hz with an interval of 1 Hz. Then, the outputting voltage from the optical accelerometer and the LDS was recorded at the same time. And the converted output acceleration signal of the LDS and the output voltage of the optical accelerometer are presented in Figure 10. The sensitivity of the accelerometer in Figure 10 measured at different frequencies is consistent with the sensitivity measured at different amplitudes in Figure 8. That is to say, the operation frequency range is 0.4–12 Hz.

**Figure 11:**
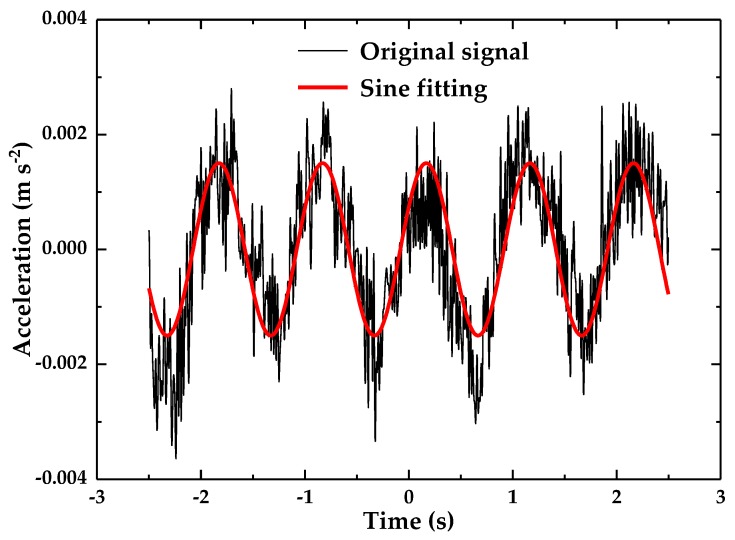
The resolution of the accelerometer.

The acceleration detection resolution and range experiments used a sine waveform with a constant frequency of 1 Hz as the excitation signal, and the amplitude of the excitation was adjusted from small to large until the accelerometer produced a clearly sinusoidal waveform. Then, the output voltage of the optical accelerometer was converted into the corresponding acceleration by dividing by the sensitivity. The experimental results are shown in Figure 11. It can be seen that the resolution of the accelerometer is 0.003 m·s^−2^ approximately. The maximum acceleration that the accelerometer can measure *a*_max_ can be calculated by:

(6) $$a_{\max}=\frac{V_{\max}}{s},$$

where *s* is the sensitivity of the accelerometer and *V*_max_ is the maximum voltage that the accelerometer can output. The maximum voltage that the accelerometer can output is 12.7 V, and its sensitivity is 1.74 V (m·s^−2^)^−1^. Therefore, the maximum acceleration that the accelerometer can measure is 7.29 m·s^−2^, and the measurement range of the accelerometer is 0.003–7.29 m·s^−2^.

**Figure 12:**
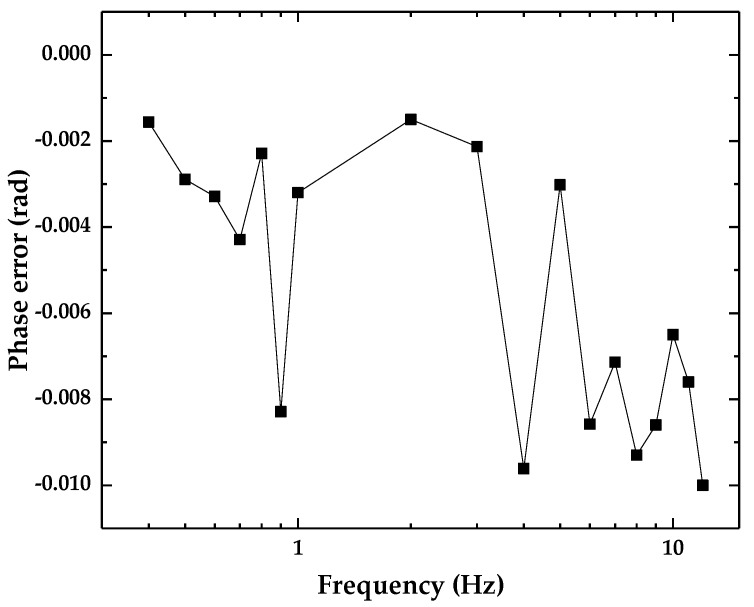
The phase error of the optical accelerometer.

Experiments were also conducted to investigate the phase-frequency performance of the accelerometer with frequencies in the range of 0.4–1 Hz with an interval of 0.1 Hz, and in the range of 1–12 Hz with an interval of 1 Hz. The LDS was configured to output analog signal of 4–20 mA, which was converted to voltage signal and collected together with the output signal of the accelerometer are collected into a computer for further processing. Origin 2017 software (OriginLab Co., Ltd., Northampton, MA, USA) was used to fit the two signals, and then the phase difference between the two fitted waveforms was calculated, which is shown in Figure 12. The experimental results show that the accelerometer has a nonlinear phase error of less than 0.011 rad, which can cause the corresponding relative error of acceleration measurement of about 0.006% (calculated by: 1−cos 0.011). This is relatively quite small and can be neglected.

### 4.4. Noise Equivalent Acceleration Measurement.

**Figure 13:**
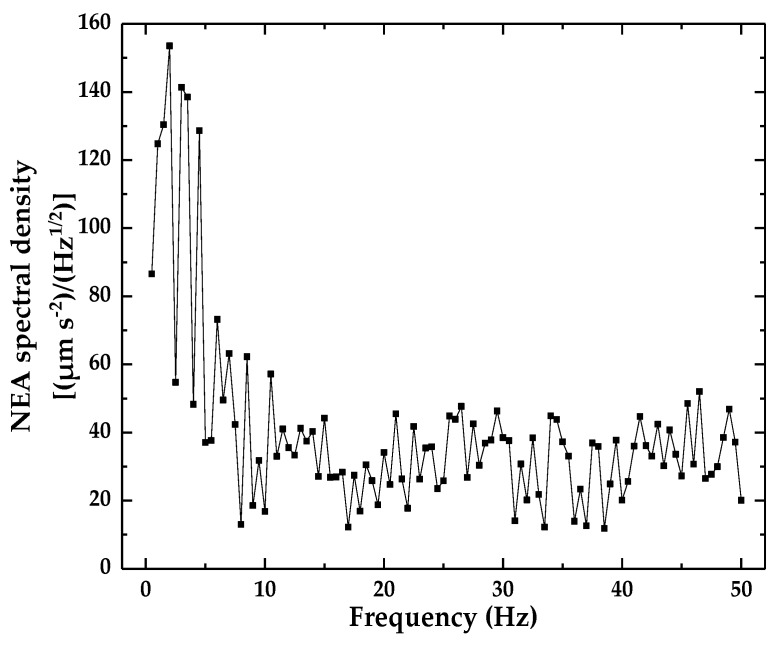
Noise equivalent acceleration (NEA) spectral density.

To measure the noise equivalent acceleration (NEA) of the designed optical accelerometer, the output voltage noise was recorded by a DAQ card when the vibration generator was turned off. The spectral density of the NEA was calculated using the sensitivity value shown Figure 8. Figure 13 plots the NEA spectral density in frequency domain. The electronic circuit of the current optical accelerometer generated an electrical noise of less than 160 (μm·s^−2^) (Hz^−1/2^) over the frequency range of 0.5–50 Hz. Considering that the NEA in this study is lower than the ones from related literature [12,13,14,15,16,19,21], the NEA in this study has been promoted visibly.

### 4.5. Signal Drift of the Optical Accelerometer

**Figure 14:**
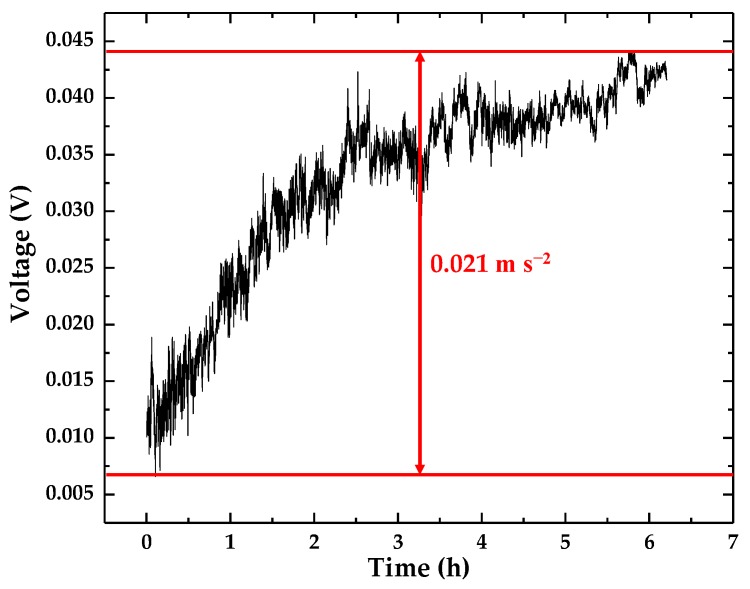
The drift of the optical accelerometer.

During the operation of the accelerometer, its accuracy is usually affected by a Gaussian laser beam, angular drift and other influencing factors. To determine the effect of these factors on the accelerometer, the accelerometer’s drift was tested for more than 6 h in a resting state. The experimental results are shown in Figure 14. From Figure 14, we can see that the 6-h drift of the accelerometer is about 37 mV, whose equivalent acceleration is 0.021 m·s^−2^.

## 5. Discussions and Conclusions

This work presents an optical accelerometer that consists of a QPD-based sensor, a leaf spring, and a seismic mass for low-frequency, low-amplitude vibration. The principle and structure of the optical accelerometer are introduced. The elastic structure is analyzed, designed, simulated, and fabricated. The experimental results show that the accelerometer has a sensitivity of 1.74 V (m·s^−2^)^−1^, the measurement range of the accelerometer is 0.003–7.29 m·s^−2^, the operating frequencies range of 0.4–12 Hz, and electrical noise of less than 160 (μm·s^−2^) (Hz^−1/2^) is found over the frequency range of 0.5–50 Hz.

The accelerometer is useful in the detection of low-frequency, low-amplitude vibration and can be used as a sensor in an active vibration isolation system. Furthermore, different elastic structures can be designed (such as by changing the shape or thickness of the leaf spring and increasing or decreasing the mass of the seismic mass), and the amplification of the signal processing circuit can be modified to widen the frequency response range of the accelerometer. The proposed system can be applied to the acceleration measurement of a broad range of vibrational fields, such as the monitoring and diagnosis of machine tools and structural health monitoring.

## Figures and Tables

**Table 1:** Summary of accelerometers of low-frequency micro-vibration. BW: bandwidth; PZT: piezoelectric; FBG: fiber Bragg grating; MEMS: micro-electro-mechanical system.

Principle	Sensitivity (V/g)	Range (g)	BW (Hz)	Noise-Density (μg Hz^−1/2^)	Study
**PZT**	9 mV/g	–	–	–	[3]
	X-0.93 mV/g	>0.04	<100	–	[4]
	Y-1.13 mV/g				
	Z-0.88 mV/g				
	15.6 mV/g	–	60–1.5 k	1.7	[5]
	2.82	–	2–500	–	[6]
**Strain**	–	0–5	<100	–	[7]
	Variable	±20,000 με	<100	70	[8]
**FBG**	*	–	0.7–20	–	[9]
	0.135	0.1–2	80–800	–	[10]
	0.362	< 0.5	1–10	–	[11]
	*	–	0–25	–	[12]
	*	0.5–1.5	20–70	–	[13]
	*	0.1–0.4	5–15	–	[14]
**MEMS**	1.2	±3	0.2–1500	0.3	[15,16]
	2	±1	0–50	70	[18]
	1	±2	0–50	140	[19]
	0.66	±2	0–100	30	[20]
	0.66/0.22	±6/±2	0–100	30	[21]
	0.66	±2	0–50	50	[22]
	1.2	±3	0–1500	0.3	[23,24]
**Optical**	12.28	<0.017 ^★^	3–24	20	[25]
	24.36	<0.0023 ^★^	3–6	–	[26]
	22.9	<0.08 ^★^	0.5–50	30	[27]
**Others**	–	–	0.2–0.4	–	[28]
	*	–	5–400	0.09	[29]
	–	–	20–140	0.048	[30]

“*” indicates that the parameter is not comparable; “–” indicates that the parameter is not mentioned; “^★^” indicates that the parameter is not mentioned, and read from the picture of the paper.

**Table 2:** Modal analysis and static analysis.

Thickness (mm)	Leaf Spring Shape	Resonant Frequency (Hz)			Statics Deformation (μm)	Elastic Coefficient (mN/μm)
		1st Order	2nd Order	3rd Order		
**0.1**	a	70.33	2156.2	2158.9	50.527	1.21
	b	20.82	24.01	24.11	577.53	0.11
	c	48	1245.6	1246.5	81.61	0.56
	d	76.02	2411.9	2414.2	43.03	1.42
**0.15**	a	121.87	135.85	136.77	16.96	3.61
	b	31.26	35.66	35.73	256.54	0.24
	c	89.35	108.73	109.57	24.92	1.96
	d	135.64	3299.2	3306.5	13.53	4.52
